# Experience Filter: Translating Heterogeneous Signals into Motion

**DOI:** 10.1002/advs.78602

**Published:** 2026-11-10

**Authors:** Ekrem Yüksel, Alihan Bakir, Jamie Paik

**Affiliations:** ^1^ Ecole Polytechnique Fédérale de Lausanne (EPFL) Reconfigurable Robotics Laboratory Lausanne Switzerland

**Keywords:** haptic feedback, human‐robot interaction, multisensorial feedback, signal‐to‐motion encoding, tactile force feedback

## Abstract

Human experience is inherently rich and multisensory, composed of intertwined visual, auditory, and kinesthetic feedback. Digital media capture only its audiovisual dimensions, omitting its mechanosensory dimensions. While full‐body (kinesthetic) haptic feedback can enrich media, no general method exists for deriving structured motion from heterogeneous signals and combining it with the audiovisual content for rendering. The Experience Filter maps multimedia signals into structured motion trajectories through a common pipeline of modality‐specific feature extraction followed by a modality‐agnostic shared encoding, recombining them into a synchronized multisensory experience: each signal is reduced to a shared descriptor space – signal intensity, dominant spectral feature, and distance from neutral axis – then mapped to a trajectory via a fixed, deterministic, training‐free encoding. Because this encoding space is hardware‐independent, the motion can be retargeted to robotic platforms of arbitrary degrees of freedom and morphology. A feature‐sensitivity analysis shows that the descriptor axes are independent and the encoding‐space topology follows from the chosen descriptors rather than being fixed. The framework is demonstrated on real‐world music and images, producing continuous, synchronized image‐audio‐motion experiences without manual authoring. The Experience Filter thus provides an analytical mapping from multimedia signals to combined image‐audio‐motion rendering, enabling expressive, user‐adjustable experience across robotic platforms.

## Graphical abstract

The **Experience Filter** is a signal‐agnostic framework with modality‐specific feature extraction and a modality‐agnostic shared encoding that maps heterogeneous media—music and images—into structured 3‐DoF motion. Each signal is reduced to three descriptors (intensity, dominant spectral feature, distance from neutral) and encoded into a hardware‐independent trajectory through a fixed, training‐free pipeline, enabling synchronized, user‐adjustable image‐audio‐motion experiences renderable on robotic platforms of arbitrary morphology.

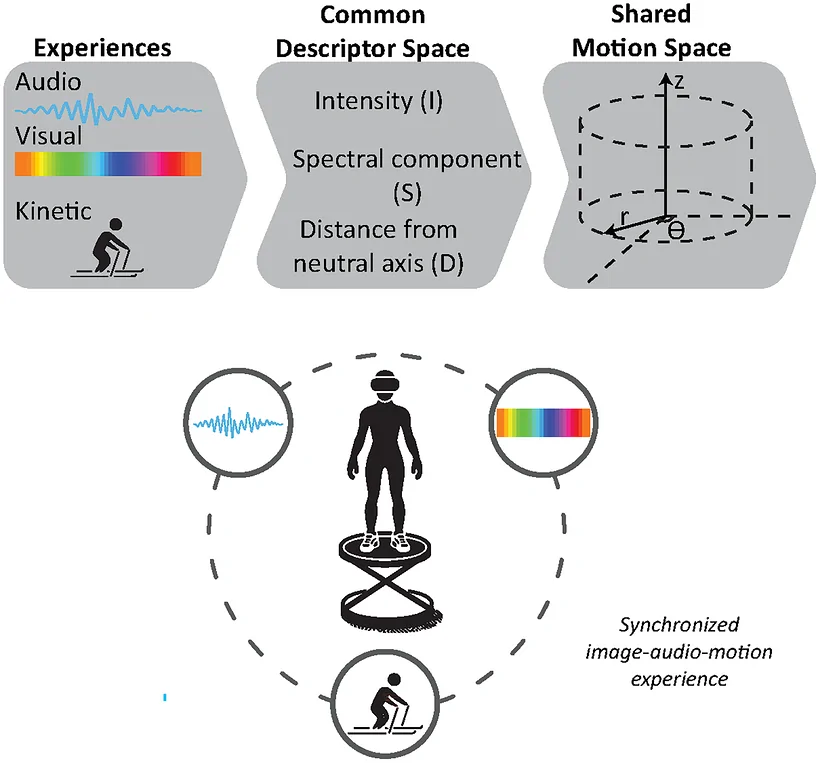

## 1 Introduction

   In music and audiovisual entertainment, vibration and tactile feedback may affect perceived quality, emotion, engagement, and presence. Merchel and Altinsoy showed that musical experience is not purely auditory: live and reproduced music can involve whole‐body vibration, reproduced vibration can contribute to the perceived quality of concert‐like listening, and perceptually optimized vibration generation may require transformations such as frequency shifting, filtering, and intensity compression [1, 2]. Related studies show that auditory cues can alter tactile roughness judgments, vibrotactile feedback can modify auditory perception, and vibro‐kinetic stimulation can increase psychological and physiological measures of affect during music listening [3, 4, 5, 6]. Nanayakkara et al. demonstrated that visual and haptic displays can enhance musical experience for hearing‐impaired users, with a haptic chair used to convey musical vibration and visual displays used to represent musical features [7]. More recent studies extend these observations to wearable and virtual environments, including musical haptic wearables for live audiences, haptic music players in VR concerts, and tactile enrichment of affective virtual environments [8, 9, 10]. In VR concerts, haptic feedback has been discussed as a way to strengthen connection, immersion, and listening engagement when the audience is not physically co‐present with performers [10]. These findings suggest that haptic augmentation can enhance media experience, but they also show that the mapping between signal and motion is not trivial. Therefore, the analysis presented here is not between perception and signal but more between the signal and resulting motion. If the motion is structured and consistent amongst different modalities, it can provide common ground for comparison.[Supplementary-material advs78602-supl-0001]

Prior work in haptic media design has addressed this mapping problem from several directions. Maggioni et al. measured the added value of haptic feedback in audiovisual media and found that haptically enhanced content can be perceived as more pleasant, unpredictable, and creative [11]. This does not mean that the media object itself becomes objectively more creative, but rather, the added tactile channel changes the user's appraisal of the media experience. It can increase liking, introduce a sense of novelty or surprise, and alter what users expect the audiovisual content to feel like as it unfolds. In musical haptics, audio‐tactile rendering includes methods for transforming musical features such as rhythm, pitch, melody, timbre, and loudness into vibrotactile stimuli, while haptic‐interface work emphasizes the need for meaningful tactile languages, objective device characterization, and physically intuitive feedback controls [12, 13, 14, 15, 16, 17]. Together, these studies show both the promise and the fragmentation of haptic media. While many systems can enhance a particular experience, none provide a general representation that can translate heterogeneous signals into physically renderable bodily motion.

The perceptual basis for such translation is supported by research on crossmodal correspondences. Crossmodal correspondences are systematic associations between features that belong to different sensory modalities, even when there is no direct physical equivalence between them. Recent works have identified associations between high pitch, small size, brightness, and elevated spatial position [18, 19]. These correspondences matter for physical rendering because they provide constraints for mapping one signal type onto another. When an auditory or visual feature is contingent with a motion cue, the user is more likely to relate the motion to the original signal as they are parts of the same event. In this sense, correspondences can enhance engagement by enabling multisensory cues to cohere as a single event, facilitating interpretation by providing intelligible qualities such as intensity, direction, weight, or energy, and fostering expectation by allowing the user to anticipate changes in rendered motion based on the variations in the source signal. In art and music cognition, music and image are linked through shared affective dimensions such as valence and arousal, musical succession has long been interpreted through motion‐like constructs such as momentum and inertia, and synesthetic or quasi‐synesthetic mappings between pitch, tonality, and color have motivated computational and artistic representations of sound as visual structure [20, 21, 22, 23, 24, 25, 26]. For a robotic interface, the key implication is that motion cues derived from music, images, or video need not be physically literal to be meaningful. A rising pitch, a brighter image region, or a stronger rhythmic accent does not require the platform to rise, tilt, or pulse in physical meaning. Nevertheless, such mappings can become meaningful when they are sufficiently consistent for users to learn, controllable to tune, and interpretable to connect the motion with the source media. This distinction is central to this work: the goal is not always physical simulation, but the construction of coherent physical cues that preserve salient features across modalities.

Robotics and machine learning have also explored the conversion of audio and other signals into motion. Such conversion is supported by mature audio‐analysis work: pitch and spectral features can be extracted from music signals, timbre and sound‐event descriptors provide perceptual information about sources and scenes, and music‐emotion research has identified computational features related to melody, harmony, rhythm, dynamics, tone color, expressivity, texture, and form [27, 28, 29, 30, 31]. Early entertainment robotics systems generated dance motions from real‐time music input by extracting beat‐related features and synchronizing predefined motion libraries [32]. Similar ideas have been extended to legged robots, where beat extraction, probabilistic motion selection, and delay compensation allow a robot to dance in synchrony with live music [33]. More recent motion generation models use deep generative architectures to synthesize 3D pose sequences from audio, text, or other driving signals. Alexanderson et al. showed that diffusion models can synthesize audio‐driven human motion such as dance and co‐speech gesture while allowing style control [34]. Zhou and Wang proposed a unified driving engine for human motion generation from natural language or audio, using a shared motion‐token representation [35]. These approaches demonstrate that heterogeneous signals can drive structured motion, but their outputs are generally high‐dimensional visual or avatar motions. They do not directly compress heterogeneous media and measured bodily motion into a small, generalizable descriptor that can be rendered by a haptic platform. More broadly, learning‐based embodied‐action models translate perceptual representations into physical actions [36], and learned fusion of complementary sensing modalities has been used for detection [37].

In this work, we introduce the Experience Filter, a signal‐to‐motion framework with a modality‐agnostic shared encoding for embodied experience rendering. The framework accepts heterogeneous inputs, including inertial recordings, video‐derived trajectories, audio, and images, and transforms them into physically renderable motion cues through a common encoding pipeline (Figure 1). This architecture supports two complementary operating modes. In the first, physically recorded experiences are reconstructed directly from measured acceleration and orientation data [38]. In the second, multimedia are transformed into motion through feature extraction inspired by how humans perceive physical signals. Rather than treating each modality independently, the framework uses a shared motion descriptor space as an intermediate experiential representation, enabling both direct motion reconstruction and expressive motion generation through the same pipeline. Extracted features and motion descriptors are encoded as three‐dimensional motion cues corresponding to tilt direction, tilt magnitude, and vertical displacement. This allows music, images, video‐derived motion, and inertial recordings to be rendered through the same physical system while remaining user‐adjustable and interpretable. In this paper, haptic feedback refers to full‐body kinesthetic feedback, that is, the motion of a platform that moves the user's body or limb.

**FIGURE 1:**
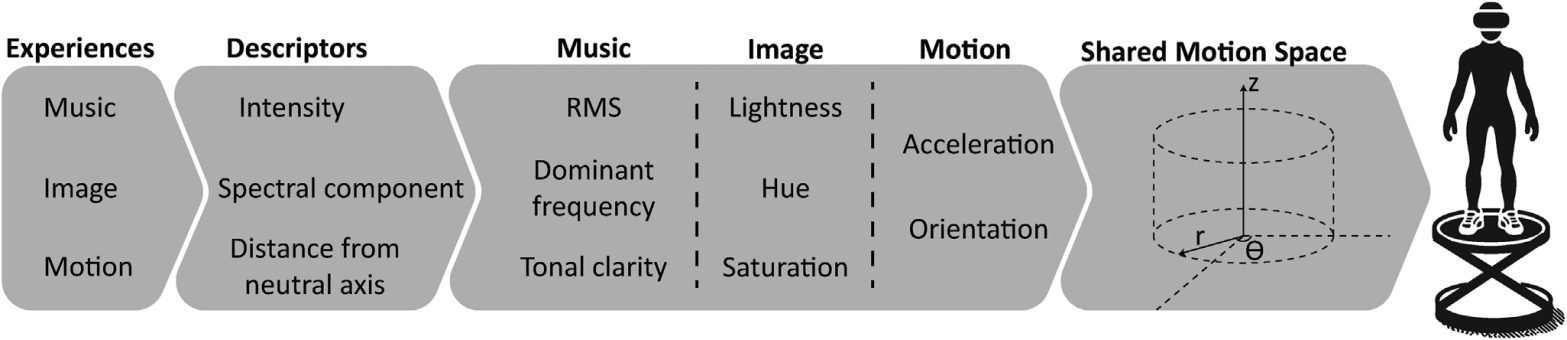
Working principle of the Experience Filter. Audio and visual signals are reduced to modality‐specific features and projected into a common descriptor space of intensity, spectral component, and distance from neutral axis, while kinetic signals provide acceleration and orientation directly. A motion‐encoding step converts both routes into the shared trajectory $M\left[k\right]=\left[\theta [k],r[k],h[k]\right]$, enabling motion on robotic platforms.

This paper makes three primary contributions. First, we propose a physical rendering framework with modality‐agnostic shared encoding capable of processing heterogeneous signal sources through a common motion‐generation pipeline. Second, we demonstrate a crossmodal motion synthesis approach in which signals without explicit kinetic information, such as music and images, can generate structured bodily motion through feature‐based encoding. Third, we introduce a shared motion descriptor representation that enables both physical recordings and digital media to be expressed within the same low‐dimensional motion space and rendered through a unified haptic platform.

In this work, the Experience Filter defines a controllable and embodied framework for transforming heterogeneous signals into motion experiences. In this sense, we demonstrate a generalized architecture for experience reconstruction and expressive motion generation across modalities.

## 2 Methods

A general signal‐to‐motion framework for generating robotic trajectories faces a key challenge: input signals do not share a common representation. Audio data represents changes in acoustic pressure over time, images capture color distribution across spatial dimensions, and inertial recordings measure acceleration over time. These data modalities differ in physical quantity, dimensionality, and, most consequentially, in whether they carry motion cues at all. Kinetic signals already carry inertial information and can be rendered on a robotic platform almost directly. In contrast, multimedia signals, such as music and images, do not carry cues that enable them to be mapped across modalities, let alone converted into motion trajectories. Hence, rendering them as motion requires an intermediate representation that bridges signal and motion. The framework must nonetheless generate a single type of output: a motion trajectory that is consistent across modalities, interpretable in terms of variations in the input signal, and usable as a reference trajectory on a robotic platform. The difficulty is therefore not extracting features from any individual signal, but constructing a representation general enough to be shared across modalities while remaining specific enough to generate motion.

To address this, we propose the Experience Filter, a signal‐to‐motion encoding framework that maps digitized signals into a motion trajectory for robotic systems. The Experience Filter extracts the modality‐specific features of a signal and encodes them into a shared encoding space (Figure 1). This bounded space serves as an intermediate representation, functioning as a bridge between signal and motion. The motion trajectory lies within this space and is therefore a reference that a platform can render within its limits, allowing otherwise incompatible signals to be processed through a common motion‐generation pipeline.

A signal, x, is processed frame by frame, yielding discrete signal units ${\left\{x_{k}\right\}}_{k=1}^{N}$. The framework translates these units into motion through feature extraction and motion encoding:

$$M\left[k\right]=E\left({\left\{F\left(x_{k}\right)\right\}}_{k=1}^{N}\right)$$

where F(·) denotes the modality‐specific feature extraction stage and E(·) denotes the motion encoding operator. The resulting discrete trajectory M[k] represents the normalized motion output of the Experience Filter. In this section, we describe the input signals and how we categorize them, the feature extraction stage, and the motion encoding stage.

### 2.1 Signal Definition and Modality Classes

Signals are measurable quantities of an experience that can be captured via electronic sensors such as cameras, IMUs, and microphones, regardless of the physical stimulus. However, not all signals carry inertial cues. A musical piece may induce dance, or a video of a dynamic scene may evoke a sense of motion in the viewer, yet neither signal carries inertial information. Any such motion is experienced by the observer at the cognitive level. More generally, raw signals, such as RGB pixel values, accelerations, or acoustic pressure, do not correspond to what humans perceive: we perceive hue and lightness rather than wavelength, and pitch and loudness rather than frequency. While perception is a more complex cognitive output, this study focuses on the low‐level perceptual features [39].

The mapping of the signals to the encoding space is determined by their content, specifically whether they contain inertial information. Signals that carry inertial cues, such as acceleration, velocity, displacement, angular velocity, or orientation, have perceptual features that already live in the motion domain and can therefore be mapped onto the encoding space almost directly. We accordingly distinguish two signal categories: kinetic and multimedia. Kinetic signals are defined by the presence of inertial cues. For example, a skier or a sailor experiences acceleration‐induced inertial cues through the mechanosensory system, including vestibular, proprioceptive, and somatosensory feedback [39].

Multimedia signals [40], on the other hand, do not inherently carry inertial information, even when the underlying physical phenomenon involves motion or vibration. An acoustic wave produced by a vibrating string is perceived as sound characterized by acoustic properties such as frequency, amplitude, and spectral content; similarly, light reflected from a moving object may visually indicate motion, yet the resulting image is characterized by lightness, shape, and color [14, 39, 41]. Neither of these stimuli carries inertial information such as acceleration or velocity. Because no inertial component is present, these signals, and consequently their features, cannot be rendered as motion directly; instead, generating motion from them requires a constructed correspondence between their features and the encoding‐space axes. Both kinetic and multimedia signals are processed by the same Experience Filter pipeline, which extracts their low‐level perceptual features and maps them onto the shared encoding space.

Within the Experience Filter framework, these signals are represented as a sequence of signal units $x_{k}$, where N is the total number of units. For temporal signals, such as audio or inertial recordings, $x_{k}$ is a fixed‐duration time frame. For spatial signals, such as images, $x_{k}$ is a spatial patch of the partitioned input.

### 2.2 Feature Extraction

A signal unit is still a raw measurement and does not by itself indicate the perceived experience. Sensory systems encode physical stimuli into modality‐specific perceptual dimensions [39, 41]. For example, skiing is strongly characterized by inertial cues and body pose, whereas music is characterized by loudness, pitch, and timbral quality [28, 30]. Regardless of the source of the physical stimuli, for each signal unit $x_{k}$, we represent these low‐level perceptual features with a modality‐specific feature vector:

$$f_{k}=F\left(x_{k}\right)$$

where F(·) is the feature extraction function, and $f_{k}$ contains the features passed to the motion encoding stage. For kinetic signals, feature extraction selects the motion variables that dominate the target experience rather than applying a fixed set of inertial features to all motion‐based signals [38].

Multimedia signals, on the other hand, do not inherently carry motion cues and therefore require an intermediate representation before motion encoding. Feature extraction provides this representation by mapping multimedia signals into a shared encoding space. We define this encoding space with three axes: the dominant spectral feature, signal intensity, and the distance from neutral. This structure is inspired by the HSL color space, where hue corresponds to dominant spectral feature, lightness to signal intensity and saturation to distance from neutral gray [39]. This organization is further motivated by prior music‐color visualization approaches that use circular tonal spaces and color‐wheel representations to relate pitch or tonal structure to hue‐like angular organization [25, 26]. In this work, these correspondences are used as design inspiration for constructing a compatible encoding space across multimedia modalities, rather than as evidence of perceptual equivalence between audio and visual features. While the three feature dimensions are chosen to capture the dominant perceptual dimensions of each modality as established in the literature [39], the encoding space is not limited to three dimensions and can be extended by incorporating additional features as needed.

The visual information is captured in red‐green‐blue (RGB) and processed as hue‐saturation‐lightness (HSL), parallel to the human color perception [39]. A sliding window of N×M pixels with stride s is applied over the RGB image, partitioning it into a sequence of patches $\left\{x_{k}\right\}$. The sliding window follows a raster path from left to right and top to bottom as the scanning baseline. Spatial averaging is performed in RGB space before HSL conversion to avoid hue distortion that would result from averaging in HSL directly.

For audio signals, the dominant perceptual dimensions are pitch, loudness, and timbre [28, 39]. Loudness corresponds to the intensity of the audio signal and is computed as the root‐mean‐square (RMS) amplitude of each frame. The pitch‐related feature dimension is estimated as the dominant spectral feature, defined as the frequency of peak spectral magnitude in the frame's STFT spectrum, then octave‐normalized relative to the standard tuning reference $A_{4}=440$ Hz [42]. In polyphonic signals, several peaks may have similar amplitudes, and the selected peak may switch between partials or sources from frame to frame. Unlike pitch and loudness, timbre is defined as the perceptual quality of sound that is neither pitch nor loudness, and is therefore a multidimensional feature [30, 39]. Within this framework, we reduce this multidimensional feature to tonal clarity, a one‐dimensional timbre‐related feature, computed from the Harmonic‐to‐Noise Ratio (HNR). The normalized HNR ranges from 0 for a noise‐like signal to 1 for a pure tone signal, providing a bounded measure analogous to saturation in the HSL space: just as saturation collapses to zero for achromatic gray, tonal clarity collapses to zero for unpitched or noisy signals, at which point the dominant spectral feature becomes ambiguous. Tonal clarity measures how much of the in‐band power lies at the harmonics of the dominant spectral peak, i.e. the pitched‐to‐unpitched dimension of timbre. It does not represent other timbral attributes such as spectral centroid, spread, flux or attack, and inharmonic sources such as bells or percussion obtain a low tonal clarity by definition. The details of feature extraction algorithms are represented in the Supporting Information.

### 2.3 Motion Encoding

After the modality‐specific processing, the feature sequence must be represented in the intermediate layer that bridges signal and motion. Here the features are converted into modality‐agnostic references that provide a hardware‐independent trajectory, which each platform's controller adapts to its own workspace and limits. The motion encoding stage maps each unit of the feature sequence, $f_{k}$, to a discrete point in the shared encoding space:

$$M\left[k\right]=E\left({\left\{f_{k}\right\}}_{k=1}^{N}\right)={\left[m_{1}\left[k\right],\cdots ,m_{n}\left[k\right]\right]}^{⊤}\in R^{n},$$

where n is the number of degrees of freedom of the robotic interface. The resulting set of points forms a trajectory reference whose temporal spacing is determined by the control loop of the specific robotic system. While n=3 in this study, the framework generalizes to higher‐DoF platforms by expanding the encoding space and the signal parsing strategy.

The Experience Filter outputs a discrete sequence of reference points, one per signal unit (every 30 ms for the audio signal with sampling rate of 44.1 kHz used here). The conversion into a continuous command (i.e., interpolation at the control rate of the platform, filtering, and limits on velocity, acceleration and jerk) depends on the workspace, actuator bandwidth and control architecture of the platform. The Experience Filter generates the reference trajectory from the signal through explicit rules and whether a reference can be followed depends on the platform.

For kinetic signals, the dominant features are inertial and yield a motion trajectory near‐directly, as demonstrated for skiing and sailing [38]. For multimedia signals, the encoding space provides a modality‐independent intermediate representation, subsequently mapped to the motion space of the robotic interface. The nature of the selected feature dimensions dictates the topology of the encoding space: cyclic features induce periodic encoding axes, whereas bounded monotonic features induce linear ones.

Because the dominant spectral feature (hue for images or chroma for audio) is cyclic for both modalities, the encoding space takes a cylindrical topology: the dominant spectral axis maps to the azimuth θ∈[0,2π), distance from neutral axis to the radial coordinate r∈[0,1], and intensity to the height h∈[0,1]. While these features and their assignment to the motion axes are the default, both are configurable rather than fixed. The resulting normalized motion trajectory is

$$M_{\mathrm{cyl}}\left[k\right]={\left[\theta [k],\;r[k],\;h[k]\right]}^{⊤}\in D^{2}\times \left[0,1\right].$$

When an alternative representation is used for the features, the encoding topology may change. For example, when dominant wavelength and log‐normalized frequency are used as the dominant spectral features for images and audio, respectively, the spectral feature dimension becomes monotonic and bounded. All three feature dimensions are then bounded and linear, and the encoding space maps to a cubic domain, with x, y, and z corresponding to the dominant spectral feature, distance from neutral axis, and intensity, respectively:

$$M_{\mathrm{cube}}\left[k\right]={\left[x[k],\;y[k],\;z[k]\right]}^{⊤}\in {\left[0,1\right]}^{3}.$$

For audio, the two topologies make different trade‐offs. The cylindrical encoding uses the chroma (pitch class) of the dominant frequency: it captures octave equivalence, so that frequencies an octave apart map to the same azimuth, but it discards absolute pitch height. The cubic encoding instead maps the log‐normalized frequency monotonically and therefore preserves pitch height, but it does not represent octave equivalence. The two are alternatives within the same framework, and the application depends on whether the chroma or pitch height should be emphasized.

## 3 Experiments

To provide a common signal‐derived representation for mapping heterogeneous signals to motion, a generalized signal‐to‐motion encoder should produce trajectories that are repeatable, structured, and interpretable. This requires features and output axes to be decoupled, so that a variation in one feature should affect only its assigned motion coordinate. For example, a change in pitch should modify only the spectral‐motion axis while leaving the intensity and distance from neutral axes unchanged. We therefore evaluated the Experience Filter at the signal‐processing and motion‐trajectory levels using both controlled and real‐world signals. The goal of this evaluation is not to assess subjective perception or perceptual congruence, but to determine whether the proposed encoding produces traceable, axis‐decoupled, and renderable motion trajectories.

We carried out a sensitivity analysis on controlled signals in which only a single feature is varied at a time, isolating one target encoding axis. These experiments show that the encoding axes are decoupled, that the nature of the selected features determines the topology of the encoding space, and that axis independence is preserved across topologies. We then applied the complete framework to real‐world, uncontrolled signals, where multiple features vary simultaneously, to show that it still produces renderable motion trajectories. This study focuses on multimedia signals; results for kinetic signals are reported in Ref. [38].

### 3.1 Feature Sensitivity and Encoding Topology

To verify that the encoding axes are independent, we vary one feature at a time while holding the others constant, isolating each component of the output $M=[m_{1},m_{2},m_{3}]$ in turn. If the axes are decoupled, sweeping a single feature should move only its corresponding output component. In the default mapping, the selected features yield a cylindrical topology, owing to the cyclic nature of the dominant spectral feature and the bounded, scalar intensity and distance from neutral features; the dominant spectral feature, distance from neutral axis, and intensity map to $M_{\mathrm{cyl}}=\left[\theta ,r,h\right]$, respectively.

We construct three controlled signals per modality, sweeping one feature while the other two are held constant. Audio is sampled at 44.1 kHz over 20 s and analyzed in 100 ms frames (4410 samples) with a Hann window at 70% overlap; the dominant frequency is searched within a 20–2200 Hz search band. The loudness and pitch sweeps use a pure sinusoidal tone (tonal clarity = 1), while the tonal‐clarity sweep combines a pure tone with white noise to vary the signal continuously from noise‐like to tonal. Because pitch class (chroma) is octave‐invariant, the encoded azimuth is determined by the note's position on the chroma circle rather than by its absolute frequency; absolute pitch height is not encoded in this topology. The pitch sweep therefore spans $C_{1}-C_{7}$, allowing the same pitch classes to repeat across multiple octaves.

The image signals of size 700 × 30 pixels are constructed to vary features on the horizontal dimension while held constant vertically. All three inputs are processed with the same sliding averaging window and stride (30×1,s=1), yielding a signal sequence ${\left\{f_{k}\right\}}_{k=1}^{700}$, where $f_{k}\in R^{3}$. Hue is swept over $\left[0^{\circ },360^{\circ }\right)$ (S = 100%, L = 127/255), saturation over 0% to 100% (H = $0^{\circ }$, L = 127/255), and lightness from 0% to 100% (H = $0^{\circ }$, S = 0%). Figure 2 shows that a change in a single feature varies only the corresponding axis while the other two remain constant; the encoding axes are therefore decoupled. At the neutral point, where tonal clarity is 0, θ becomes undefined: the pitch class becomes perceptually ambiguous as tonal clarity vanishes, just as hue is undefined at zero saturation. This corresponds to the singularity of the cylindrical representation as r→0, as in the HSL color space.

**FIGURE 2:**
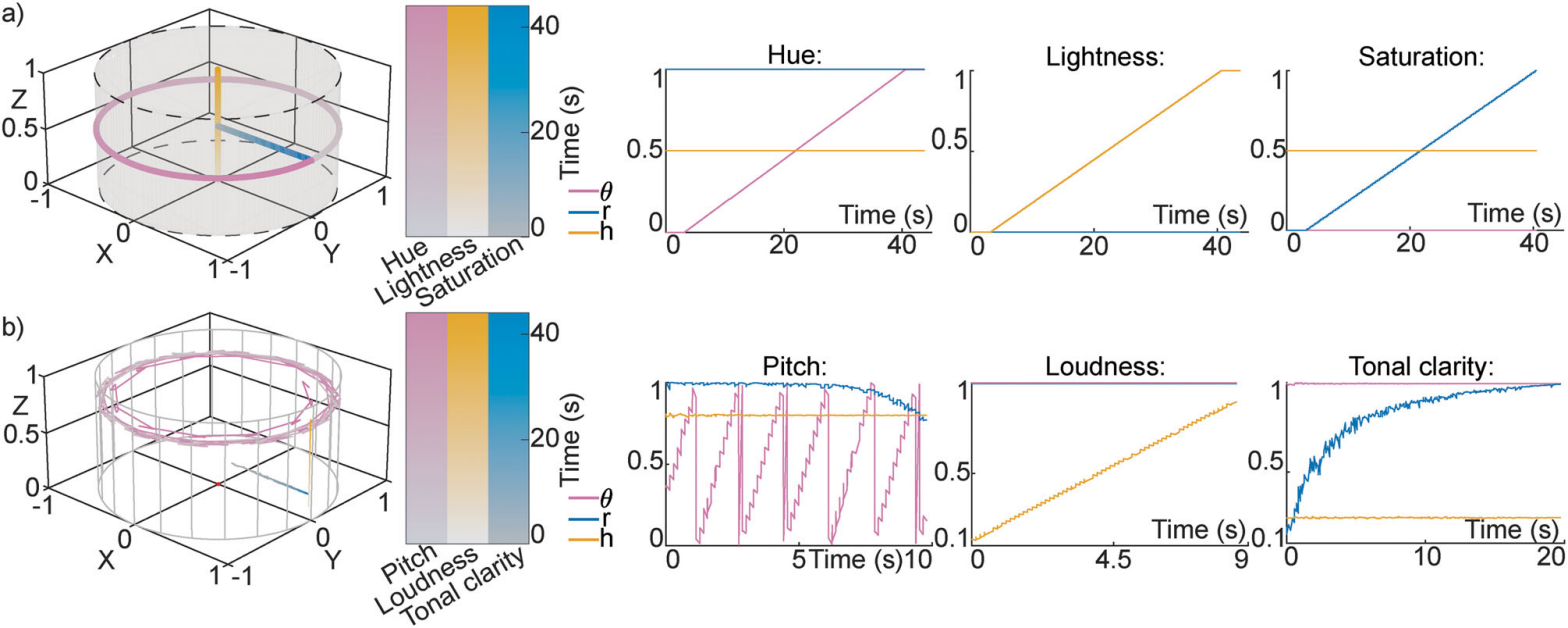
The feature sensitivity test for the default mapping. The default mapping yields a cylindrical topology. The sensitivity analysis for (a) images and (b) audio shows that the encoding axes are decoupled.

To show that the encoding topology is determined by the selected features, and that the framework is not tied to a fixed feature set or topology, we repeat the sensitivity analysis using alternative spectral measures while retaining the same intensity and distance from neutral features. We replace hue and pitch class, which are cyclic by nature, with bounded, linear measures: dominant wavelength and log‐normalized frequency, respectively. Since we are concerned with signals as perceived by humans, these measures are bounded by the perceptual range, i.e., the visible spectrum (380–720 nm) for images and the audible range (20–20 kHz) for sound. The resulting spectral dimensions are bounded and monotonic and therefore yield a cubic encoding topology, ${\left[0,1\right]}^{3}$, rather than the cylindrical topology $D^{2}\times \left[0,1\right]$, giving the output $M_{\mathrm{cube}}={\left[x,y,z\right]}^{⊺}$.

Of the two alternative spectral measures, dominant wavelength requires additional handling. The dominant wavelength is obtained by projecting the mean color from the white point onto the spectral locus, using ProPhoto RGB color space because of its wide gamut. Non‐spectral colors, such as magenta and pink, lie on the line of purples and therefore have no dominant wavelength. Conventionally, they are represented by their complementary wavelength. However, this would assign them to the green region of the normalized wavelength axis, despite their chromatic adjacency to violet and red. We therefore map violet‐ and red‐adjacent non‐spectral colors to 0 and 1, respectively, using a split complementary wavelength of $\lambda_{\mathrm{split}}=530\;\mathrm{nm}$. Because hue is a closed loop, no bounded linear axis can represent it both continuously and one‐to‐one. The split therefore introduces a discontinuity in the cubic encoding: two similar non‐spectral colors on opposite sides of $\lambda_{\mathrm{split}}$ are mapped to 0 and 1. The cylindrical encoding, in which hue is cyclic, has no such discontinuity. Non‐spectral colors have no dominant wavelength, so any value assigned to them is a convention. Further details of dominant wavelength computation as well as its visualization are given in the Supporting Information.

For audio, the spectral peak is searched within the audible range 20Hz−20kHz, and the resulting dominant frequency is log‐normalized over the same range. To span the full range of the alternative spectral features, we construct a pure sinusoidal signal whose frequency sweeps from 20 Hz to 20 kHz. For images, we use a color sequence in which the extracted dominant wavelength increases from violet to red across the visible spectrum. The remaining controlled signals and processing parameters are kept unchanged from the cylindrical condition. Figure 3 shows that varying a single feature still changes only its corresponding encoding axis, demonstrating that the encoding axes remain decoupled even when different spectral features are selected.

**FIGURE 3:**
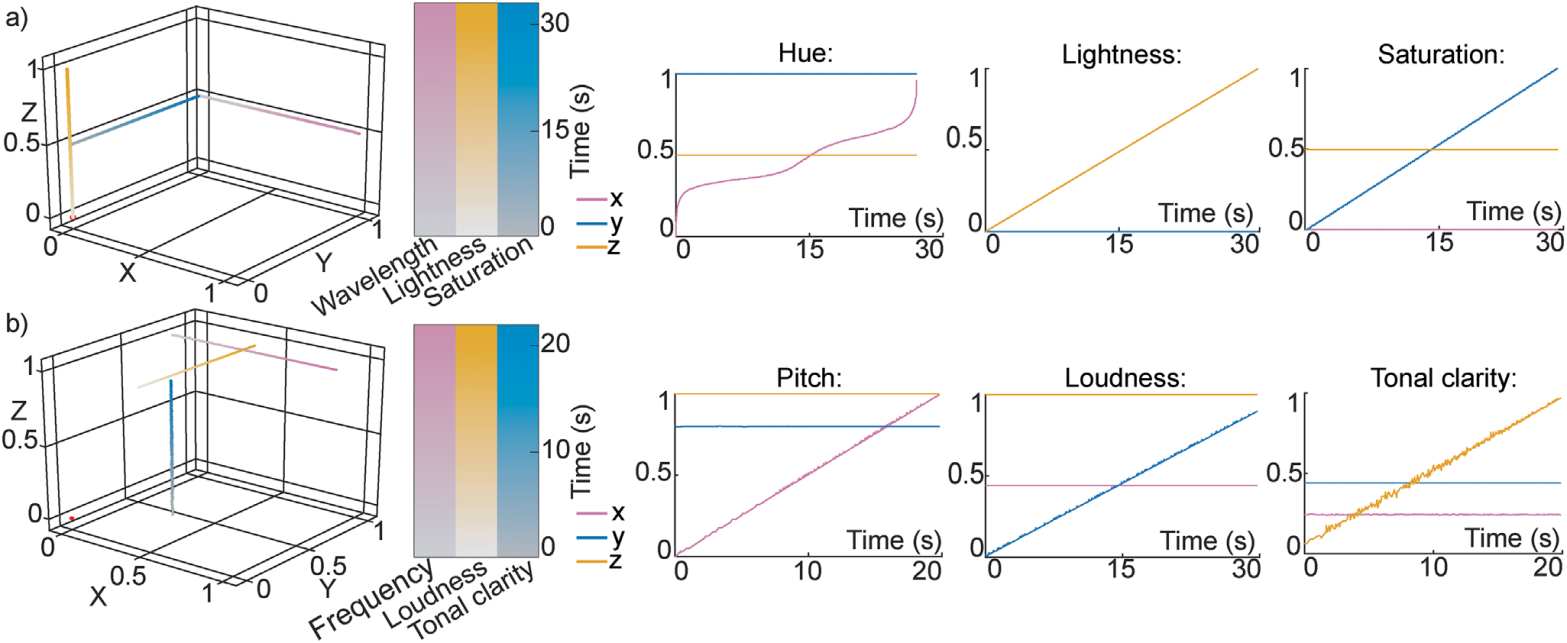
The feature sensitivity test with the alternative spectral features, which yield cubic topology. The sensitivity analysis for (a) images and (b) audio shows that the encoding axes are decoupled and independence of the axes still holds even with a new set of features.

### 3.2 Real‐World Signals

After verifying feature sensitivity and topology dependence with controlled inputs, we evaluate the complete pipeline on real‐world, uncontrolled signals under the default cylindrical encoding. Unlike the controlled inputs, where a single feature is varied at a time, real‐world signals contain simultaneous changes in spectral feature, intensity, and distance from neutral. These examples therefore test whether the Experience Filter produces continuous, structured output when multiple feature dimensions vary together.

For audio, the same frame length, overlap, and feature‐extraction parameters as in the controlled experiments are used. For images, a sliding averaging window sized to approximate a $2^{\circ }$ foveal angle [39] for a 24‐inch 1080p screen viewed from 30 cm is applied; this corresponds to a 38×38 pixel window with stride s=11, giving approximately 70% overlap between neighboring patches. To convert the two‐dimensional image into a one‐dimensional signal, the window traverses the image in a raster pattern inspired by reading: it moves from left to right along each row and then proceeds from top to bottom. The resulting sequence of patch features forms a time series that is passed to the Experience Filter in the same way as the other input signals. In contrast to the controlled signals, these inputs contain simultaneous variation across all three feature dimensions. The resulting trajectories, shown in Figure 4, are continuous and structured, demonstrating that the framework produces renderable motion without manually designed motion commands.

**FIGURE 4:**
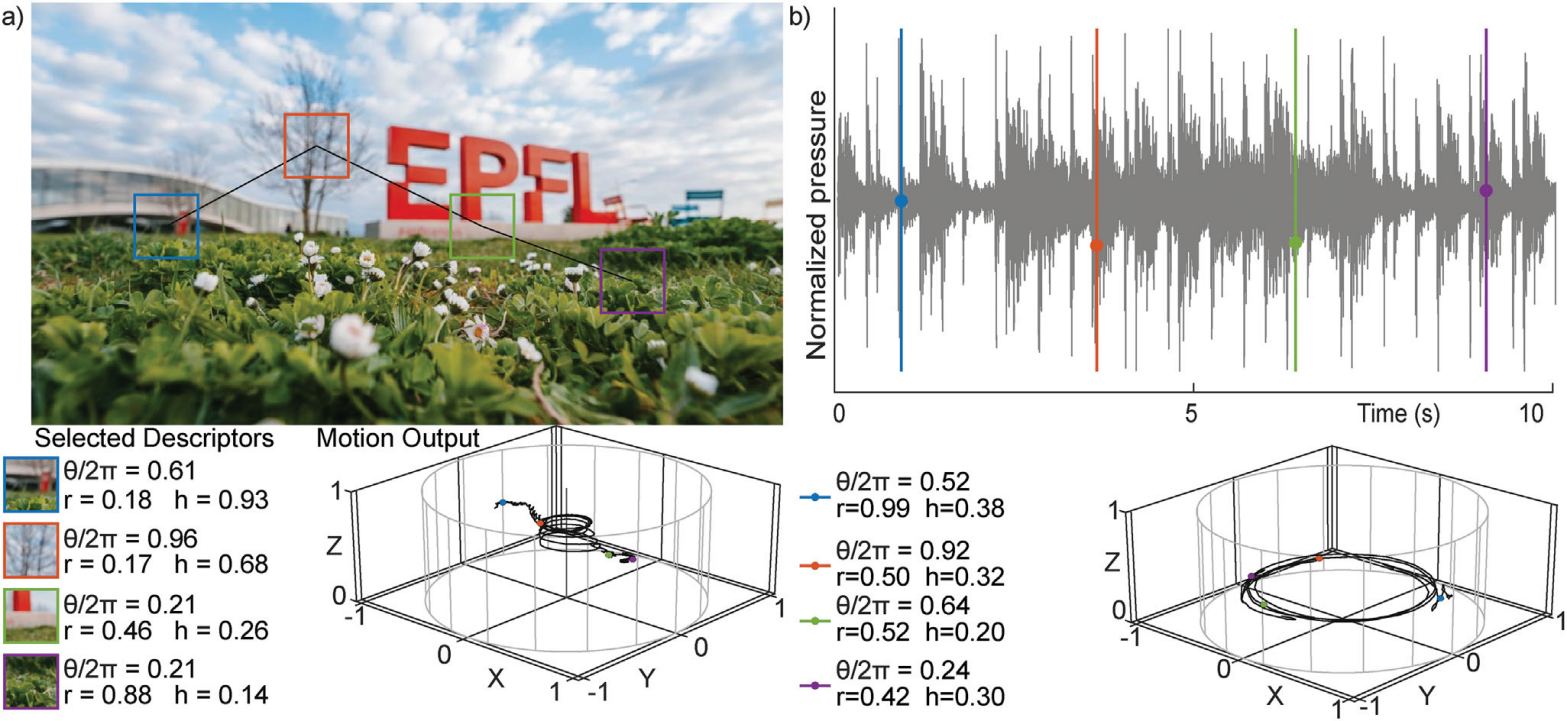
Real‐world signals as input for Experience Filter using the default cylindrical encoding. For each input, the figure shows the original signal, encoded descriptor values at four representative samples indicated in blue, orange, green, and purple, respectively, and the resulting output trajectory, M[k] within the encoding space. Results are shown for (a) a photograph and (b) a 10 s music excerpt. Unlike the controlled signals, these real‐world inputs contain simultaneous variations in intensity, dominant spectral feature, and distance from neutral axis. The Experience Filter maps these co‐varying features into continuous, structured, and renderable motion trajectories.

### 3.3 Robotic Motion Rendering

The Experience Filter outputs a set of reference points in a bounded and normalized encoding space that is independent of any particular device. In principle, this reference can be rendered on any robotic platform whose workspace can contain the scaled encoding space. To test whether this holds on an actual physical system, we render the real‐world signals of Figure 4 on a commercially available 6‐DoF robotic arm (RM65, RealMan Robotics). We choose a manipulator for three reasons. First, the three‐dimensional encoding space maps directly onto the Cartesian space of its end effector, so no modification on the dimensions is required. Second, unlike OriDEX [38], whose workspace is close to a cylinder, a manipulator is a general‐purpose device that is not designed around the encoding space. Third, the arm provides its own inverse kinematics and controller and reports its end‐effector position with millimeter accuracy, so no additional motion tracking is required during execution.

The Experience Filter runs on one PC and streams each reference point $\left[\theta [k],r[k],h[k]\right]$ over UDP through a direct wired connection to a second PC that controls the arm. On the second PC, a platform's controller converts each reference point into an end‐effector position. The controller maps the origin of the encoding space to the initial end‐effector position, $p_{0}$, at the start of each run. To ensure that every point in the reference sequence stays within the workspace of the arm, it scales the space to a cylinder of 100 mm radius and 200 mm height.

The controller converts the output of the Experience Filter from cylindrical to Cartesian coordinates and passes the resulting x,y,z references to the arm as end‐effector position commands through the manufacturer's API. The controller of the arm then tracks these references, using its inverse kinematics to reach each end‐effector position. The arm reports its end‐effector position at 50 Hz.

Figure 5 compares the reference and measured end‐effector positions for the music excerpt and the image over 30 s of motion each. In both cases, the manipulator cannot follow the trajectory to its full extent, so the motion of the arm is attenuated and lags behind the reference. The root‐mean‐square error of the position is 48.5 mm for the music excerpt and 15.6 mm for the image. Yet, as illustrated in Figure 5, the manipulator preserves the trend of the Experience Filter trajectory.

**FIGURE 5:**
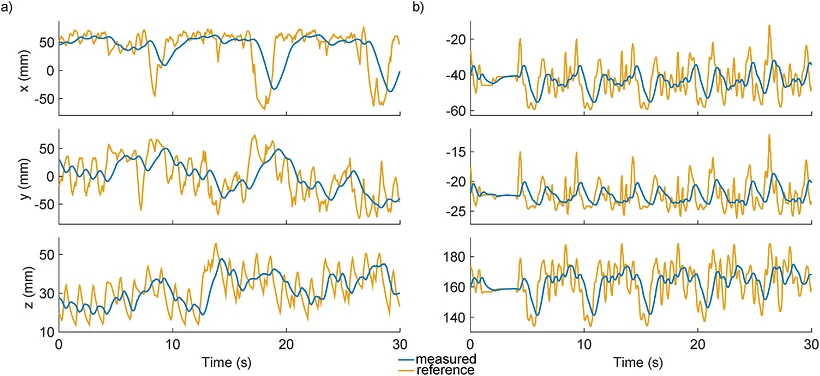
Physical rendering of Experience Filter trajectories on a robotic arm. The motion trajectory M[k] is converted to Cartesian coordinates, scaled into the robot workspace, and used as the reference end‐effector position (orange) for a 6‐DoF RealMan RM65 robotic arm; the measured end‐effector position is shown in blue. Results are shown for (a) the music excerpt and (b) the photograph of Figure 4, over 30 s of motion each, with the x, y, and z components in the top, middle, and bottom rows. Positions are given relative to the origin of the cylindrical encoding space, with unit radius scaled to 100 mm and unit height to 200 mm.

We measure the similarity with Spearman's rank correlation coefficient ρ [43]. Once we compensate for a lag of 0.72 s for the music excerpt and 0.38 s for the image, the root‐mean‐square error of the position decreases to 28.1 and 8.9 mm, and ρ becomes 0.76, 0.82, and 0.75 for the music excerpt and 0.78, 0.76, and 0.81 for the image along x, y, and z, respectively. The lag is larger for the music excerpt, whose reference contains higher and faster change of θ, while the end effector velocity does not exceed 76 mm/s. Therefore, the arm follows the reference trajectory, although it cannot match it exactly.

## 4 Discussion

The experiments demonstrate that the proposed framework converts signals from different modalities into structured 3‐DoF trajectories through the same signal‐processing pipeline. The feature sensitivity analysis shows that the encoding axes are independent. Moreover, the repeated analysis with alternative feature selection shows the decoupling holds, indicating the independence is a property of the encoding space itself rather than a fixed topology or set of features. The experiments show that the topology of the encoding space follows the nature of the selected features.

When we apply the framework to real‐world signals, in which intensity, spectral content, and distance from neutral vary simultaneously, the Experience Filter produces continuous and structured trajectories without any manual intervention or tuning. While in this study we do not address whether the generated motion aligns with human cross‐modal perception, the decoupling of the encoding axes provides structurally interpretable motion that is consistent with the varying features. Because each descriptor drives exactly one motion coordinate, the contribution of every feature to the trajectory is traceable by construction. For example, as each piano key is played in turn, the resulting motion is traceable and predictable, advancing around the chroma circle in the cylindrical encoding and moving monotonically with pitch in the cubic one. Notably, this predictability is what establishes the bridging layer between non‐kinetic signals and motion.

Earlier music‐driven motion systems take a different approach. A legged robot can dance to music by extracting the beat in real time and selecting movements from a user‐generated motion library at each beat [33], so the motion is retrieved from a predefined set rather than derived from the signal itself. More recent audio‐ and text‐driven models instead rely on deep generative architectures trained on large motion corpora to synthesize high‐dimensional pose or avatar sequences [34, 35]. The Experience Filter does neither: it is analytical, involving no motion library, no training, and no learned parameters. Each trajectory is computed deterministically from the input signal through explicit feature extraction and a fixed encoding operator, so its outputs are reproducible and compact, compressing signals to a low‐dimensional descriptor suited to a motion platform rather than to visual animation.

Because the output is an abstract motion trajectory rather than a device‐specific trajectory, the same descriptor sequence can drive robotic platforms with different kinetics and degrees of freedom, provided their workspace can contain a scaled variant of the encoding space. The cylindrical encoding maps directly onto the roll, pitch, and height of the OriDEX platform [38]. The same trajectory can be projected onto the planar coordinates of a pantograph mechanism by dropping the height axis, or onto the spatial coordinates of a drone, letting it trace the trajectory through space. The framework extends to platforms with more than three degrees of freedom by enlarging the descriptor space and adapting the parsing strategy to the available motion channels.

When we render the real‐world trajectories on a robotic arm, the arm follows the trend of the reference, with a slight lag. The lag is due to the actuation limitations of the robotic platform. We expect a faster platform to follow the same reference more closely, whereas a slower one requires the platform's controller to adapt the trajectory to its own limits. In this work, we evaluate the Experience Filter at the signal‐processing and motion‐trajectory levels and do not assess how the generated motion is perceived. Whether this motion is perceived as natural, intuitive, or congruent with the source signal requires further user studies. Future work should characterize the physical interaction forces between the user and the platform.

The features are intentionally low‐dimensional: distance from neutral reduces the multidimensional timbre to a single scalar, and the HSL features do not capture composition, depth, or semantic content in an image. The framework is also signal‐agnostic only at the encoding stage; each new modality requires its own feature‐extraction front end to be designed and implemented so that its signals map into the descriptor space, which can be a laborious task. Furthermore, the current design encodes the instantaneous value of each feature rather than its evolution over time or space, even though the perception of an experience depends not only on instantaneous values but also on how a signal is built up over time. Content‐aware processing, for instance following the contours of an image rather than averaging local patches, or capturing rhythm and attack in audio, would address this and extend the framework toward richer, more immersive motion. Because the encoding axes are independent, such features can be added as further axes without disturbing the existing coordinates.

Beyond rendering, the controllability of the encoding, and hence of the resulting motion output, suggests a further use: because motion excitation can be varied independently along each axis, the platform could serve as an instrument for studying how motion maps to psychological state, for example whether low‐frequency, low‐amplitude motion produces calming effects while stronger excitation drives arousal. Together, these directions build toward expressive, user‐adjustable experience rendering grounded in a single signal‐to‐motion architecture.

## 5 Conclusion

We introduced the Experience Filter, a deterministic signal‐to‐motion framework that translates signals through modality‐specific feature extraction followed by a shared motion‐encoding stage. For audio and image signals, the framework represents each signal using three low‐level feature dimensions: intensity, dominant spectral feature, and distance from neutral. These dimensions are mapped into structured motion trajectories, with n = 3 in the present implementation and extension to higher‐DoF systems achieved by expanding the encoding space and parsing strategy. Controlled feature‐sensitivity analysis verified the intended one‐to‐one correspondence between features and motion coordinates and showed that coordinate decoupling is preserved across both cylindrical and cubic encoding topologies. The results further demonstrated that the topology of the encoding space follows the cyclic or monotonic nature of the selected features rather than being fixed in advance. Passing real‐world signals through the framework yielded continuous motion trajectories. When these trajectories were rendered on a commercially available 6‐DoF robotic manipulator, the arm followed their trend, although it could not match their full dynamics. This confirms that the Experience Filter generates reference motion trajectories for robotic devices regardless of their morphology, limited only by the capacity of the device. Unlike predefined motion libraries or learning‐based motion generation methods, the Experience Filter computes each trajectory analytically through explicit feature extraction and motion encoding operators. These results establish the Experience Filter as an interpretable intermediate representation between heterogeneous non‐kinetic signals and robotic motion, providing a foundation for rendering on further robotic platforms and for perceptual evaluation.

## Author Contributions

E.Y., A.B., and J.P. designed and developed the Experience Filter framework. E.Y., A.B., and J.P. designed the study and interpreted the results. E.Y. and A.B. drafted the manuscript and prepared the figures. J.P. revised the manuscript and drafted the abstract.

## Funding

This work was supported by the Swiss National Science Foundation Fund for Approaching Tangible Reality with Reconfigurable Robots project (204819).

## Conflicts of Interest

The authors declare no conflicts of interest.

## Supporting information


**Supporting File 1**: advs78602‐sup‐0001‐SuppMat.pdf.

**Supporting File 2**: advs78602‐sup‐0002‐VideoS1.mp4.

**Supporting File 3**: advs78602‐sup‐0003‐VideoS2.mp4.

## Data Availability

Data sharing not applicable to this article as no datasets were generated or analyzed during the current study.
