# Supplementary material for: Experience Filter: Translating Heterogeneous Signals into Motion
Source: Adv Sci (Weinh). 2026 Nov 10:e78602. Online ahead of print. doi: 10.1002/advs.78602 (PMC13752583; doi:10.1002/advs.78602)
Supplement: Supplementary file 1 [file ADVS-9999-e78602-s003.pdf]

Feature Extraction Algorithms

This section details the feature extraction function $F(\cdot)$ for the two multimedia modalities used in this work, audio and images. For each signal unit x_k, an audio frame or an image patch, the function returns the three feature dimensions that populate the encoding space of Section 2.2: signal intensity, the dominant spectral feature, and the distance from neutral.

Audio Intensity

Intensity is the root-mean-square (RMS) amplitude of the frame, normalized by that of a full-scale sinusoid, $\frac{1}{\sqrt{2}}$, so that a full-scale tone maps to unit intensity. For a frame $x_k = \{x_n\}_{n=1}^{L}$ of L samples,

$$\mathrm{RMS}_k = \sqrt{\frac{2}{L}\sum_{n=1}^{L} x_n^2},$$

clipped to $[0, 1]$ and assigned to the intensity axis (h, z).

Audio Dominant Spectral Feature

Each frame is analyzed with the short-time Fourier transform (STFT), giving a Hann-windowed magnitude spectrum $|X(f_i)|$ with bin spacing $\Delta f = \dfrac{f_s}{N_{\text{fft}}} = 10\,\text{Hz}$, where $N_{\text{fft}} = 4410$ is the frame length (a 100 ms frame at $f_s = 44.1\,\text{kHz}$). The dominant frequency is the spectral peak within the analysis band $[f_{\min}, f_{\max}]$. The peak bin i^* is refined to sub-bin precision by fitting a cubic polynomial $p(x) = ax^3 + bx^2 + cx + d$ to the four magnitude samples at offsets $x \in \{-1, 0, 1, 2\}$ about i^*; the refined offset δ is the root of $p'(x) = 3ax^2 + 2bx + c = 0$ nearest the peak, with parabolic interpolation over the three central samples as a fallback. The dominant frequency is then

$$F_{\text{peak}} = f_{i^*} + \delta\,\Delta f.$$

In the default cylindrical encoding, the peak is reduced to a cyclic pitch class by octave normalization relative to the standard tuning reference $f_{\text{ref}} = 440\,\text{Hz}$ (A_4):

$$\theta = 2\pi \log_2\left(\frac{F_{\text{peak}}}{f_{\text{ref}}}\right)$$

In the cubic encoding, the same peak is expressed on a bounded, monotonic log-frequency axis over the audible range $[f_{\min}, f_{\max}] = [20,\ 20000]$ Hz:

$$z = \frac{\log_2\big(F_{\text{peak}}/f_{\min}\big)}{\log_2\big(f_{\max}/f_{\min}\big)} \in [0, 1].$$

Audio Distance from Neutral: Tonal Clarity

The distance from neutral is the tonal clarity, a timbre-related feature derived from the harmonic-to-noise ratio (HNR): the fraction of the frame's in-band spectral power that lies at the harmonics of F_{peak}. The harmonics are at kF_{peak}, $k = 1, \ldots, K$, where K is the number of harmonics below the Nyquist frequency $f_s/2$, capped at 15. Around each harmonic the power spectrum $P(f) = |X(f)|^2$ is summed within a band of half-width $\Delta = \max(0.1\,F_{\text{peak}}, 25\,\text{Hz})$, and the tonal clarity is this harmonic power divided by the total in-band power:

$$C_k = \frac{\sum_{f\in\mathcal{H}} P(f)}{\sum_{f_{\min}\le f\le f_{\max}} P(f)} \in [0, 1],$$

where $\mathcal{H}$ collects the frequencies within $\pm\Delta$ of any harmonic. A value of 1 means a pure tone, with all power at the harmonics, and 0 a noise-like signal. Tonal clarity drives the distance-from-neutral axis (radius r); when the signal is unpitched, $C_k \to 0$ and the feature collapses to the neutral axis ($r = 0$), where the dominant spectral feature is undefined, just as hue is undefined at zero saturation.

Image Color Features

Each image patch x_k of $N \times M$ pixels is averaged in RGB space to a single triplet $\bar{c} = (R, G, B) \in [0, 1]^3$; averaging is performed in RGB rather than HSL. The mean color is converted to hue, saturation, and lightness, with $\Delta = \max(R, G, B) - \min(R, G, B)$:

$$L = \frac{\max(R, G, B) + \min(R, G, B)}{2}, \qquad S = \begin{cases} 0 & \Delta = 0, \\ \dfrac{\Delta}{1 - |2L - 1|} & \Delta > 0, \end{cases}$$

$$H = \frac{1}{6} \begin{cases} \big((G - B)/\Delta\big) \bmod 6 & R = \max(R, G, B), \\ (B - R)/\Delta + 2 & G = \max(R, G, B), \\ (R - G)/\Delta + 4 & B = \max(R, G, B), \end{cases} \qquad H \in [0, 1).$$

Lightness L is assigned to the intensity axis and saturation S to the distance-from-neutral axis. Under the cylindrical topology the cyclic hue is the dominant spectral feature, with $\theta = 2\pi H$; under the cubic topology the dominant spectral feature is replaced by the dominant-wavelength axis of the following section.

Image Dominant Wavelength

For the cubic topology, the cyclic hue is replaced by a bounded, monotonic dominant-wavelength axis computed from the mean color through its CIE chromaticity. The mean RGB triplet is converted to CIE XYZ using the configured RGB color space (ProPhoto RGB, chosen for its wide gamut) and white point W, and then to chromaticity coordinates (x, y).

The dominant wavelength follows the Helmholtz construction [44, 45] (Fig. S1): a ray is drawn from the white point W through the color's chromaticity point $C = (x, y)$, and its intersection with the spectral locus gives the dominant wavelength λ_d. Non-spectral colors, the magentas and purples on the line of purples, have no such intersection on the near side, so the construction instead returns the negative complementary wavelength λ_c, where the ray extended in the opposite direction meets the locus. These geometric operations are performed with the `colour` library [46] (`RGB_to_XYZ`, `XYZ_to_xy`, `dominant_wavelength`).

The signed wavelength is normalized to a bounded axis in $[0, 1]$, as summarized in Algorithm S1. Spectral colors ($\lambda_d > 0$) are mapped linearly over the visible range $[\lambda_{\min}, \lambda_{\max}] = [380, 720]$ nm, from violet (0) to red (1). Non-spectral colors are collapsed to the nearest chromatic end by a hard split at $\lambda_{\text{split}} = 530$ nm: red-adjacent magentas ($\lambda_c < \lambda_{\text{split}}$) map to 1 and violet-adjacent purples to 0, so that the line of purples joins the red and violet ends rather than folding back into the green region of the axis. This split is the only discontinuity of the cubic encoding. A smooth blend across it would instead place the purples on the values of spectral colors (e.g. magenta on green). Achromatic colors, for which the dominant wavelength is undefined, are mapped to the neutral value 0.5, mirroring hue being undefined at zero saturation.

Sensitivity Analysis and Independence

As the Experience Filter maps each descriptor to its own output coordinate, the encoder itself cannot couple the axes. What can introduce errors or coupling is the feature extraction in front of it. Figure S2 therefore tests the complete pipeline in two ways. Figure S2(a) tests whether the outputs for real-world signals remain close to their original values when noise is added to the input and (b) tests whether the output axes remain independent when only one feature changes.

For Figure S2(a), we added noise to the real-world inputs from Section 3 and re-ran the full feature extraction. For the image, Gaussian noise was added to every pixel and colour channel with the noise

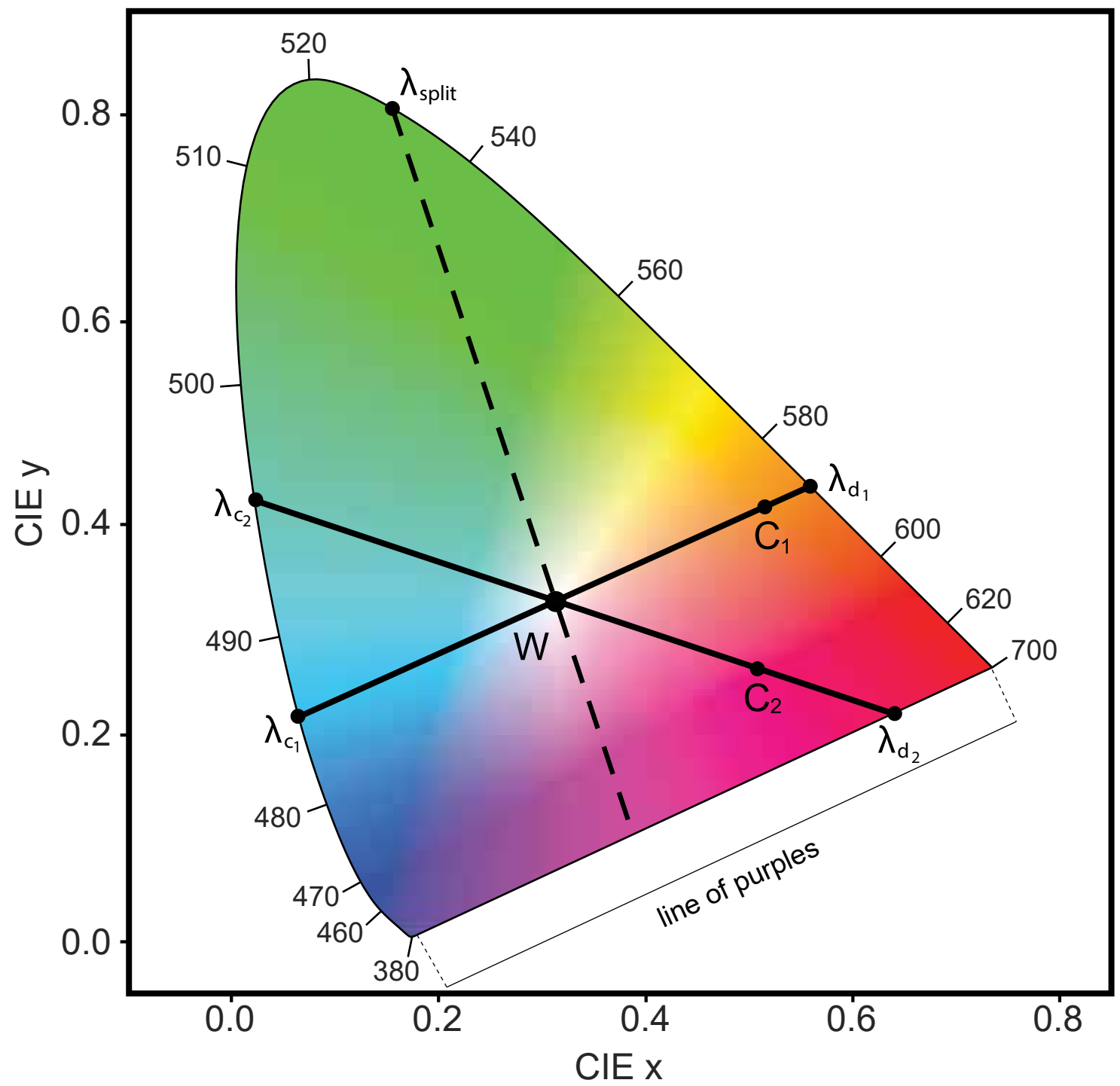

Figure S1: **Helmholtz construction of the dominant wavelength on the CIE 1931 xy chromaticity diagram.** A ray from the white point W through a colour's chromaticity point is intersected with the spectral locus; the forward intersection gives the dominant wavelength and the opposite intersection the complementary wavelength. The spectral colour C_1 yields a dominant wavelength λ_{d1} on the locus, with complementary λ_{c1}. The non-spectral colour C_2 lies on the line of purples, where the ray has no spectral dominant (λ_{d2}); it is therefore represented by its complementary wavelength λ_{c2} on the locus. The dashed split line at $\lambda_{\text{split}} = 530\,\text{nm}$ separates red-adjacent non-spectral colours, mapped to the red end of the axis, from violet-adjacent ones, mapped to the violet end.

Algorithm S1 Dominant-wavelength axis from mean RGB

Require: mean color $\bar{c} \in [0,1]^3$; RGB color space with white point W; bounds $\lambda_{\min}, \lambda_{\max}$; split λ_{split}
Ensure: dominant-wavelength axis $a \in [0,1]$

```
if max(c̄) − min(c̄) < ε then                                   ▷ achromatic color
    return 0.5
end if
XYZ ← RGB_to_XYZ(c̄, color space, W)
(x, y) ← XYZ_to_xy(XYZ)
if XYZ invalid or (x, y) ≈ W then
    return 0.5
end if
λ_d ← dominant_wavelength((x, y), W)                  ▷ ray–locus intersection, Fig. S1
if λ_d > 0 then                                                   ▷ spectral color
    return clip((λ_d − λ_min)/(λ_max − λ_min), 0, 1)
else                                                          ▷ non-spectral color
    λ_c ← |λ_d|
    return 1 if λ_c < λ_split else 0
end if
```

standard deviations from 0.02 to 0.4 of the full scale, corresponding to noise powers of 0.43%, 2.6%, 9.6%, 33% and 98% of the original image. For the music, Gaussian noise was added at noise powers of 0.01%, 0.1%, 1%, 10% and 100% of the signal power. For every window or frame, we compared the outputs for the noisy signal with those for the original signal. The cells of the matrices give the RMS error of each axis, over all windows or frames.

The outputs degrade gradually as the noise power increases. At a noise power of 10%, the RMS error is 0.016, 0.056 and 0.007 for the image, and 0.043, 0.082 and 0.020 for the music for θ, r and h respectively.

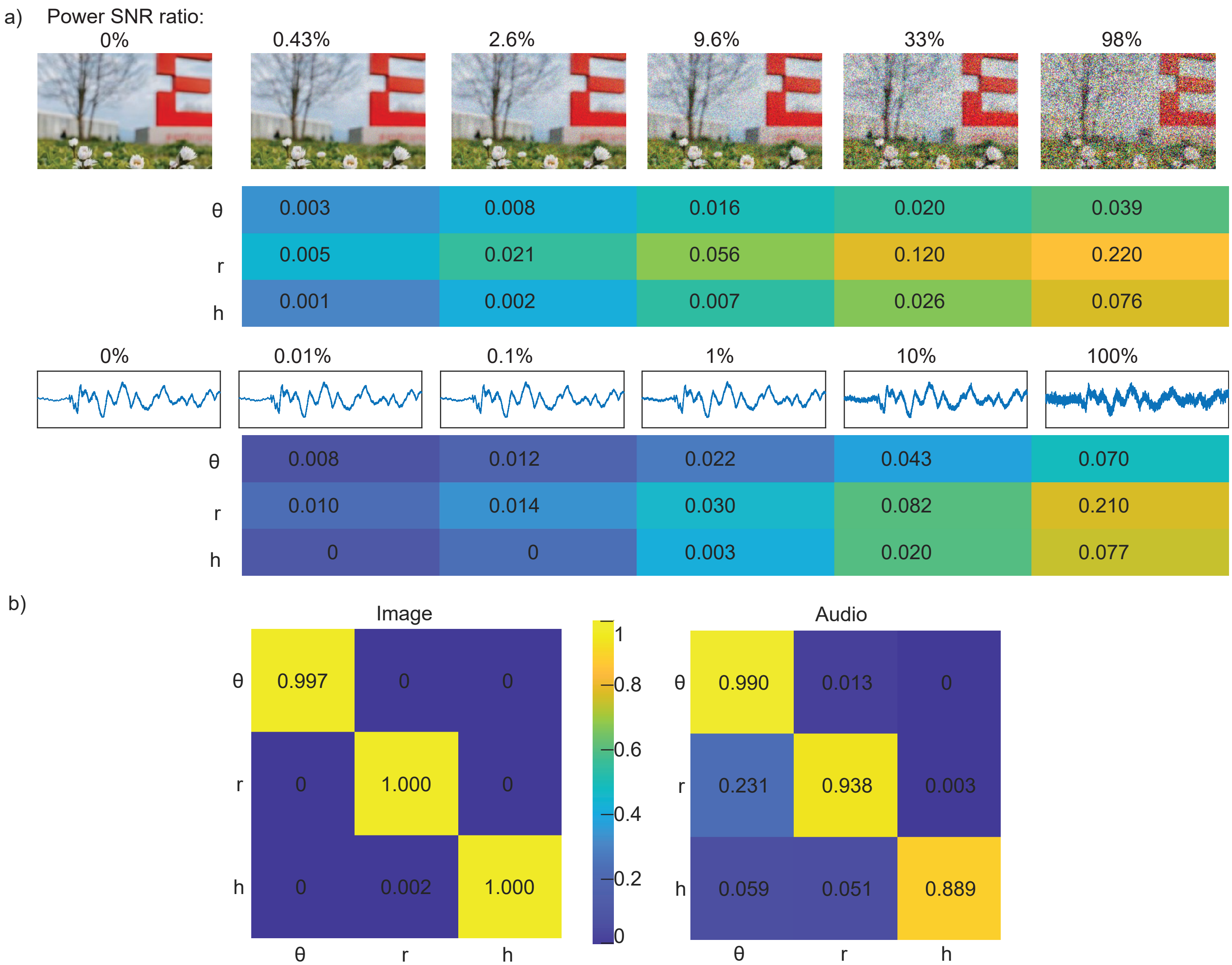

Figure S2: **Robustness to noise and independence of the encoding axes.** (a) Robustness to noise for the real-world image (top) and music (bottom). Noise power is given above each column as a percentage of the signal power (0% is the original/clean input). Each row of a matrix is an output axis (θ, r, h), and each cell is the RMS error of that axis compared to its value for the clean signal. (b) Independence of the encoding axes on generated signals in the cylindrical topology. Each column is the feature that is swept (the other two are held constant), and each row is the range covered by the output axis. A matrix with ones on the diagonal and zeros elsewhere means the axes are completely independent.

At a noise power equal to the signal power, the maximum axis error is around 0.2. The θ change at this level corresponds to about 14° of hue for the image and 7% of an octave for the music.

For Figure S2(b) we used the controlled signals of the feature-sensitivity experiment from Section 3 in the cylindrical topology. For images, hue was swept over $[0°, 360°]$ at 100% saturation, saturation from 0% to 100% at a hue of 0°, and lightness from 0% to 100% with 0% saturation. For audio (44.1 kHz, 20 s, 100 ms frames with 70% overlap), pitch was swept over $C_1 - C_7$, loudness was swept for a pure tone, and tonal clarity was swept by mixing a pure tone with white noise. For each sweep, we recorded the three output coordinates and computed the range they cover (99.9th percentile of the samples, which is practically the full range), in normalized units. Entry (i, j) of a matrix is the range of output axis i when feature j is swept. A swept axis ideally covers its full range (diagonal close to 1), and the other axes stay constant (off-diagonal close to 0).

For images, the matrix is the identity: the diagonal entries are approximately 1, and the largest off-diagonal entry is 0.002. For audio, the diagonal entries are around 0.9. Most off-diagonal entries are

comparatively small.
